# Effects of maternal folic acid supplementation during the second and third trimesters of pregnancy on neurocognitive development in the child: an 11-year follow-up from a randomised controlled trial

**DOI:** 10.1186/s12916-021-01914-9

**Published:** 2021-03-10

**Authors:** Aoife Caffrey, Helene McNulty, Mark Rollins, Girijesh Prasad, Pramod Gaur, Joel B. Talcott, Caroline Witton, Tony Cassidy, Barry Marshall, James Dornan, Adrian J. Moore, Mary Ward, J. J. Strain, Anne M. Molloy, Marian McLaughlin, Diane J. Lees-Murdock, Colum P. Walsh, Kristina Pentieva

**Affiliations:** 1 Nutrition Innovation Centre for Food and Health (NICHE), School of Biomedical Sciences, Ulster University, Coleraine, BT52 1SA Northern Ireland, UK; 2 Northern Health and Social Care Trust, Causeway Hospital, Coleraine, Northern Ireland, UK; 3 Intelligent Systems Research Centre, Ulster University, Derry~Londonderry, Northern Ireland, UK; 4 Department of Computer Science, BITS Pilani, Dubai campus, Dubai, United Arab Emirates; 5 Institute of Health and Neurodevelopment, College of Health and Life Sciences, Aston University, Birmingham, UK; 6 Psychology Research Institute, Ulster University, Coleraine, Northern Ireland, UK; 7 Royal-Jubilee Maternity Service, Belfast, Northern Ireland, UK; 8 School of Environmental Sciences, Ulster University, Coleraine, Northern Ireland, UK; 9 School of Medicine, Trinity College, Dublin, Ireland; 10 Genomics Medicine Research Group, School of Biomedical Sciences, Ulster University, Coleraine, Northern Ireland, UK

**Keywords:** Prenatal folic acid, Pregnancy, Randomised controlled trial, Child cognition, Neuronal function, Wechsler Intelligence Scale for Children, Magnetoencephalographic brain imaging

## Abstract

**Background:**

Maternal folic acid (FA) supplementation before and in early pregnancy prevents neural tube defects (NTD), but it is uncertain whether continuing FA after the first trimester has benefits on offspring health. We aimed to evaluate the effect of FA supplementation throughout pregnancy on cognitive performance and brain function in the child.

**Methods:**

Follow-up investigation of 11-year-old children, residing in Northern Ireland, whose mothers had participated in a randomised trial of Folic Acid Supplementation in the Second and Third Trimesters (FASSTT) in pregnancy and received 400 μg/day FA or placebo from the 14th gestational week. Cognitive performance (Full Scale Intelligence Quotient, Verbal Comprehension, Working Memory, Perceptual Reasoning, and Processing Speed) was assessed using the Wechsler Intelligence Scale for Children. Neuronal function was assessed using magnetoencephalographic (MEG) brain imaging.

**Results:**

Of 119 mother-child pairs in the FASSTT trial, 68 children were assessed for neurocognitive performance at 11-year follow-up (Dec 2017 to Nov 2018). Children of mothers randomised to FA compared with placebo scored significantly higher in two Processing Speed tests, i.e. symbol search (mean difference 2.9 points, 95% CI 0.3 to 5.5, *p* = 0.03) and cancellation (11.3 points, 2.5 to 20.1, *p* = 0.04), whereas the positive effect on Verbal Comprehension was significant in girls only (6.5 points, 1.2 to 11.8, *p* = 0.03). MEG assessment of neuronal responses to a language task showed increased power at the Beta (13–30 Hz, *p* = 0.01) and High Gamma (49–70 Hz, *p* = 0.04) bands in children from FA-supplemented mothers, suggesting more efficient semantic processing of language.

**Conclusions:**

Continued FA supplementation in pregnancy beyond the early period currently recommended to prevent NTD can benefit neurocognitive development of the child. MEG provides a non-invasive tool in paediatric research to objectively assess functional brain activity in response to nutrition and other interventions.

**Trial registration:**

ISRCTN ISRCTN19917787. Registered on 15 May 2013.

**Supplementary Information:**

The online version contains supplementary material available at 10.1186/s12916-021-01914-9.

## Background

Folate is essential in early life, with conclusive evidence that periconceptional supplementation with folic acid (FA; the synthetic vitamin form) is effective in preventing the first occurrence [1] and recurrence [2] of neural tube defects (NTD). This evidence has led to recommendations that are in place worldwide for women to take FA from before conceiving until the end of the first trimester. What is less clear is whether continued FA supplementation after the first trimester of pregnancy can confer longer term health benefits to the child. Emerging evidence however shows that the period of rapid growth and development of the foetal brain occurring in later pregnancy is particularly sensitive to maternal folate concentrations [3]. Also, myelination of the brain, which is most intensive from mid-gestation to the second year of life [4] and essential for cognitive development as it protects nerve axons and facilitates communication between neurons, may be particularly vulnerable to deficiency of folate [5]. Several studies have investigated the association of maternal folate in pregnancy with neurocognitive development in the child [6]. Over 40 years ago, Gross et al. showed that children born to mothers diagnosed with folate-related megaloblastic anaemia in the third trimester of pregnancy had abnormal neurodevelopment and lower intellectual abilities, compared to infants born to mothers with optimal folate status [7]. Decades later, a longitudinal transgenerational study found that optimal maternal folate status during later pregnancy was associated with better cognitive performance in the child at 9–10 years [8]. Another study of note used magnetic resonance imaging (MRI) of the child’s brain along with cognitive tests and found that maternal folate deficiency in later pregnancy was associated with, not only lower language and visuospatial abilities, but also reduced brain volumes in children aged 6–8 years [9].

A major limitation in the aforementioned studies is that they are observational and thus, by design, cannot demonstrate that maternal folate nutrition is causatively linked with cognitive outcomes in the offspring [6]. More robust evidence was however provided in our recent follow-up study of children whose mothers had participated in a randomised trial of Folic Acid Supplementation in the Second and Third Trimesters (FASSTT) [10] and showed improved psychometrically measured cognition at 3 years and word reasoning (verbal IQ) at 7 years in the children of mothers randomised to receive FA in pregnancy [11]. The latter study, and almost all previous research in this area, involved psychological tests to assess neurodevelopment in children, whereas direct measurements of neuronal activity in the brain have rarely been employed. Magnetoencephalography (MEG) is a non-invasive neuroimaging modality that measures the magnetic fields associated with neuronal currents generated by the brain and may thus provide a robust platform for investigating neurodevelopment in children [12]. Therefore, in the present FASSTT Offspring trial, we aimed to evaluate the effect of FA supplementation during pregnancy on cognitive performance and MEG-assessed brain functioning in the child at 11 years. Our hypothesis was that the higher verbal IQ previously found in the 7-year-old children of mothers randomised to receive FA in pregnancy would remain evident in these children at 11 years, as measured by both cognitive testing and MEG assessment.

## Methods

### Study population

This study was conducted as a follow-up investigation of children at 11 years whose mothers participated in the FASSTT trial in pregnancy, as described in detail elsewhere [10]. Briefly, healthy pregnant women, aged 18–35 years with a singleton pregnancy and who had taken 400 μg/day of FA in the first trimester (as per current guidelines in the UK), were recruited from antenatal clinics in Northern Ireland before the 14th gestational week (GW). Women were excluded from participation in the trial if they had a previous NTD-affected pregnancy or were taking medications known to interfere with folate or related B-vitamin metabolism. Among the inclusion criteria were taking FA supplements sometime in the 1st trimester at the recommended use of 400 μg/day; this automatically excluded any mother taking no FA or taking higher dose FA. At the start of the second trimester, pregnant women were stratified into tertiles of plasma homocysteine (a functional marker of folate status) and subjects in each stratum were randomised to receive FA (400 μg/day) or placebo until the end of pregnancy (i.e. a total intervention 26 weeks). FA supplements were distributed (in 7-day pillboxes) to participants every 4 weeks and an overall participant compliance rate of 93% was estimated from records of unused tablets.

**Fig. 1:**
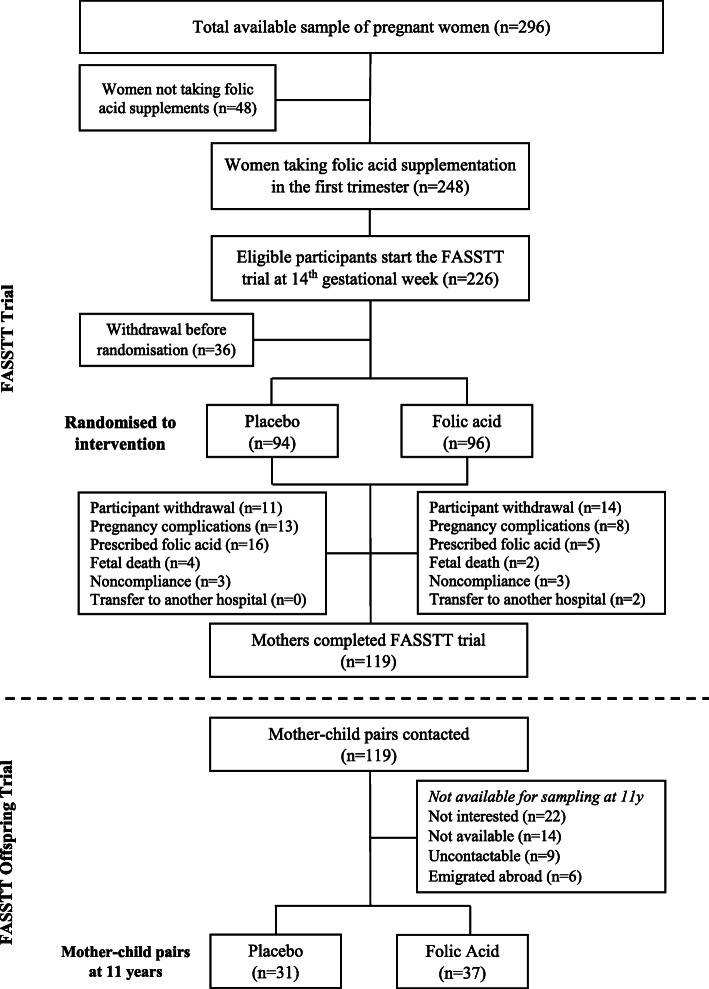
Diagrammatic protocol of the FASSTT Offspring trial at 11 years

The current FASSTT Offspring study was conducted from December 2017 to November 2018, when the child was 11 years old (Fig. 1). Mother-child pairs were recruited by invitation letter to attend an appointment at the Nutrition Innovation Centre for Food and Health (NICHE), Ulster University. All efforts were made to recruit the maximum number of participants from the original FASSTT trial. If current contact details were not readily available from our research records, the details were traced through health records. Those who failed to attend for appointment were offered an alternative date, up to a maximum of five appointments. A participant information sheet was provided to the mother, whilst a child-friendly version was provided to explain the study to the child. In compliance with ethical requirements, signed informed consent from the mother and assent from the child were obtained at the beginning of the appointment. Ethical approval was granted by the Office for Research Ethics Committees Northern Ireland (ORECNI Ref: 12/NI/0077) and the study was registered (www.isrctn.com/ISRCTN19917787).

### Scale for intellectual development evaluation

Cognitive performance of the child was assessed by the Wechsler Intelligence Scale for Children 4th UK Edition (WISC-IV) using Q-interactive™ software (Pearson Education Ltd., UK) and performed using iPads [13]. The WISC-IV assessment is suitable for children from 6 years to 16 years and 11 months and provides composite scores for specified cognitive IQ domains—Verbal Comprehension, Perceptual Reasoning, Working Memory and Processing Speed comprising 15 subtests—which are combined to give one composite score that represents the child’s general intellectual ability (Full Scale IQ). The assessment of each child lasted 90–120 min and the entire test battery was completed in one session. To ensure an ideal environment for assessment of the child, the room was bright, ventilated and free from distraction and interruptions. The same trained researcher (AC) conducted the assessments of all children and was blinded to the treatment allocation of the mother during the FASSTT trial.

### Magnetoencephalography acquisition and analysis

In a sub-sample of participants, MEG brain imaging was performed in the Northern Ireland Functional Brain Mapping (NIFBM) facility at the Intelligent Systems Research Centre, Ulster University. Before beginning the MEG task, participants were given verbal instructions and watched a child-friendly video to explain MEG, as produced by Aston University [14]. Once in the MEG machine, the children were encouraged to remain as still as possible and were provided with earphones, asked to listen carefully and to try to understand the sentences. Eye movements and heartbeat rhythm were monitored by bipolar electrodes. Head movement was recorded using head-position indicator (HPI) equipment with coils placed on the scalp. For each participant, head shape and HPI coil position were defined using a 3D digitiser (Fastrak, USA). As no MRI scans were performed for individual brain structures, in order to provide anatomically informed MEG analysis, the HPI digitisation points were realigned to the individual head shape for each participant and then registered to the recorded MEG data.

Based on our previous findings showing higher word reasoning scores (verbal IQ) at 7 years in the children of FASSTT trial participants randomised to receive FA in pregnancy [11], and observational evidence linking maternal folate deficiency in pregnancy with lower language abilities in the child [9], a language task was selected to test the underlying neurophysiological basis of language processing in this study using MEG. For this purpose, we adapted a high-level semantic language task [15] to compare the difference in neural responses to two types of sentences, which are rendered either congruent or incongruent at the presentation of the final word (e.g. ‘The baby was thirsty and wanted a drink of milk/fire’, where ‘fire’ or ‘milk’ renders the sentence semantically incongruent, or congruent, respectively): (Additional File 1: Methods). The MEG responses were recorded with the Elekta Neuromag 306-channel MEG system (using the operating conditions as detailed in Additional File 1: Methods). The band power in response to the language paradigm was obtained from 116 brain regions and averaged within six standard spectral bands: Big band [1–48 Hz], Theta [4–8 Hz], Mu [8–12 Hz], Beta [13–30 Hz], Low Gamma [30–48 Hz] and High Gamma [49–70 Hz].

### Data collection: related covariates

#### General health and lifestyle measures

Health and lifestyle information was gathered from the mother using a detailed questionnaire that included information on socioeconomic status which was mapped using house number, street name, postcode, town, and the Land and Property Services Ordnance Survey of Northern Ireland POINTER Geo-referencing database [16]. After verifying the address information, all participants were geo-referenced and linked to an ‘area deprivation’ score based on the Census Output Area in which they lived and using data from the Northern Ireland Multiple Deprivation Measure 2010 (MDM) score [17].

Standardised calibrated equipment was used to measure height (portable stadiometer, SECA, Birmingham), head and waist circumference (non-stretchable tape) and weight and body fatness (TANITA, TBF-410, Netherlands). BMI *Z*-scores were calculated using World Health Organization AnthroPlus software for assessing growth in children and adolescents of 5–19 years [18].

#### Blood sampling and B-vitamin biomarkers

As previously detailed, non-fasting blood samples were collected from the mother at 14th and 36th GW, and the umbilical cord blood was collected at delivery [10]. For the current study, a paediatric phlebotomist obtained non-fasting blood samples from the child at 11 years. All blood processing was carried out within 4 h of collection and stored at − 80 °C until analysis. Samples were analysed for serum and RBC folate, serum vitamin B12 and plasma homocysteine using standard methods, and riboflavin status was determined using the erythrocyte glutathione reductase activation coefficient (EGRac) assay, as described elsewhere [19]. For each assay, quality controls were provided by the repeated analysis of pooled samples covering a wide range of values. Intraassay and interassay CVs were ≤ 8.2% for RBC folate, ≤ 10.4% for serum vitamin B12, ≤ 2.5% for plasma homocysteine and ≤ 2.8% for EGRac. Genotype for the common C677T polymorphism in the gene encoding methylenetetrahydrofolate reductase (MTHFR) was identified using polymerase chain reaction amplification, followed by HinF1 digestion.

#### Dietary analysis

Dietary intakes were evaluated using a 4-day food diary (which included 2 weekdays and 2 weekend days) in combination with a food frequency questionnaire (FFQ), a method previously validated for folate and related B-vitamin intakes against biomarker values, as described in detail elsewhere [20]. Vitamin supplement use was also recorded. Nutrient intakes were analysed using the Nutritics nutritional software package [21], which was customised to include the most recent nutritional content of fortified food products.

### Statistical analysis

Confirmation that the sample size remained appropriate for cognitive performance testing was based on the assessment scores in the WPPSI-III test from these children previously sampled at 7 years [11]. A sample size of 37 children in each group was estimated to detect a significant difference in cognitive performance between the children of mothers randomised to FA versus placebo during pregnancy, with a power of 80% at *α* = 0.05.

Statistical analysis was performed using the Statistical Package for the Social Sciences software (version 25.0; SPSS UK Ltd., Chertsey, UK). Data not normally distributed were log-transformed before analysis. Differences between placebo and treatment groups were analysed using independent *t* tests for linear variables and *χ*^2^ tests for categorical parameters. Raw cognitive scores were automatically standardised for the child’s age at time of testing, and appropriate age-specific reference intervals were applied in adherence with test protocols. Analysis of covariance (ANCOVA) was used to test for differences in cognitive test variables and MEG power (semantic processing efficiency) between treatment groups, with adjustment for relevant confounding factors, including socioeconomic status (MDM score) [22] and child’s sex [23]. In all analyses, a 2-sided *p* value < 0.05 was considered significant.

## Results

### Study population

Of the 119 participants in the FASSTT trial [10], 68 mother-child pairs completed the FASSTT Offspring trial at 11 years, representing a 57% response rate. Maternal characteristics, including biomarker responses to intervention with FA or placebo during pregnancy, were similar for mothers participating in the current study as compared with the total sample in the original trial (Additional File 1: Table S1). No adverse events were reported at any time during the original FASSTT trial or the follow-up studies of mother-child pairs.

Maternal, neonatal and child health characteristics are reported in Table 1, showing no significant differences in general characteristics between the FA and placebo groups. In mothers during pregnancy, there were also no significant differences between the treatment groups in serum or RBC folate at the 14th GW (pre-intervention), but after FA intervention for 26 weeks, these folate biomarkers were significantly higher in the FA group in maternal (at 36 GW) and in cord blood samples (Table 2). In children at 11 years, there were no significant differences between the treatment groups in dietary intakes or biomarkers of folate or metabolically related B-vitamins. Mean dietary folate and B-vitamin intakes compared favourably with European Food Safety Authority (EFSA) population reference intake (PRI) values for children 11–14 years [24], and B-vitamin biomarker concentrations were in good agreement with those reported in population-based surveys in children from Norway [25], the UK [26] and the USA [27].

**Table 1:** General characteristics of mother-child pairs from the FASSTT Offspring trial

	Placebo (*n* = 31)	Folic acid (*n* = 37)
Maternal characteristics at 14th GW		
Age, years	28.1 (26.6, 29.6)	29.7 (28.5, 30.8)
BMI, kg/m^2^	25.2 (23.7, 26.7)	25.5 (23.6, 27.3)
Smoker, No. (%)	2 (6)	5 (14)
Duration of FA use at sampling, weeks^a^	13.3 (10.5, 16.0)	12.7 (10.3, 15.0)
Iron supplement use, No. (%)	6 (19)	11 (30)
Parity, No.	0.7 (0.4, 1.0)	0.9 (0.6, 1.2)
Caesarean section, No. (%)	9 (29)	10 (27)
Education, years	19.2 (18.3, 20.2)	19.6 (18.8, 20.4)
Married, No. (%)	24 (80)	33 (92)
Owner of dwelling, No. (%)	24 (80)	30 (83)
Rural dweller, No. (%)	16 (52)	22 (59)
Socioeconomic status (MDM score)^b^	21.9 (17.0, 26.8)	18.7 (15.3, 22.0)
Neonatal characteristics		
Gestational age, weeks	40.2 (39.6, 40.7)	39.8 (39.4, 40.2)
Sex, male, No. (%)	15 (48)	16 (43)
Birth weight, g	3503 (3329, 3677)	3461 (3290, 3633)
Birth length, cm	51.1 (50.2, 52.0)	50.9 (50.1, 51.7)
Head circumference, cm	34.5 (34.0, 35.0)	34.6 (34.1, 35.1)
Apgar score at 1 min	8.4 (7.9, 8.8)	8.4 (8.0, 8.8)
Apgar score at 5 min	8.9 (8.8, 9.1)	8.9 (8.7, 9.1)
Breastfed, No. (%)	15 (48)	16 (43)
Child characteristics (11 years)		
Age, years	10.8 (10.7, 10.8)	10.8 (10.7, 11.0)
Siblings, No.	1.9 (1.5, 2.3)	1.7 (1.5, 2.0)
Weight, kg	39.3 (35.8, 42.8)	37.7 (35.3, 40.1)
Height, cm	147.1 (144.8, 149.3)	146.7 (144.0, 149.3)
BMI for age *Z*-score^c^	0.15 (− 0.33, 0.62)	− 0.03 (− 0.45, 0.38)
Waist circumference, cm	71.1 (67.3, 74.9)	68.2 (65.6, 70.7)
Head circumference, cm	55.2 (54.2, 56.2)	54.7 (54.0, 55.4)
Body fatness, %	19.6 (15.8, 23.3)	19.2 (16.5, 21.9)

Continuous measures presented as mean (95% CI), unless otherwise indicated; compared by independent t-test. Categorical measures compared using Pearson’s chi-square

^a^The duration of FA use refers to the total number of weeks, from initiating FA supplements to time of sampling at the 14th gestational week of pregnancy

^b^Northern Ireland Multiple Deprivation Measure 2010 (MDM). This is a measure of socioeconomic area-based deprivation and comprises 7 domains, each developed to measure a distinct form of deprivation: income, employment, health, education, proximity to services, living environment and crime

^c^*Z*-scores for BMI were calculated using the WHO AnthroPlus software [18]

**Table 2:** Folate and related B-vitamin status of mother-child pairs from the FASSTT Offspring trial

		Placebo (*n* = 31)	Folic acid (*n* = 37)	*p v*alue
**Mothers during pregnancy**				
Preintervention (14th gestational week)				
Dietary intakes				
Energy, MJ/day		8.463 (7.631, 9.296)	7.995 (7.468, 8.522)	0.31
Dietary folate equivalents, μg/day		371 (306, 437)	388 (329, 447)	0.71
B vitamin status				
Serum folate, nmol/L		50.5 (43.1, 57.9)	49.9 (42.8, 57.1)	0.91
RBC folate, nmol/L		1055 (839, 1271)	1271 (1059, 1482)	0.15
Serum B12, pmol/L		236 (209, 264)	233 (206, 259)	0.85
Plasma homocysteine, μmol/L		6.1 (5.6, 6.6)	6.1 (5.6, 6.6)	0.96
*MTHFR* 677TT genotype, No. (%)		4 (13)	5 (14)	0.91
Postintervention (36th GW)				
Serum folate, nmol/L		22.4 (15.9, 28.8)	52.9 (45.8, 60.0)	< 0.001
RBC folate, nmol/L		907 (722, 1042)	1864 (1650, 2078)	< 0.001
Serum B12, pmol/L		169 (151, 188)	175 (152, 198)	0.98
Plasma homocysteine, μmol/L		7.2 (6.5, 7.9)	6.3 (5.8, 6.8)	0.04
**Neonates at birth**				
Serum folate, nmol/L		68.2 (57.8, 78.5)	105.2 (93.4, 117.0)	< 0.001
RBC folate, nmol/L		1512 (1272, 1752)	2216 (1856, 2575)	0.001
Serum B12, pmol/L		286 (230, 342)	259 (216, 302)	0.42
Plasma homocysteine, μmol/L		9.6 (7.7, 11.4)	10.4 (8.9, 11.9)	0.35
*MTHFR* 677TT genotype, No. (%)		3 (13)	4 (13)	0.99
**Children at 11 years**				
Vitamin supplement user, No. (%)		9 (30)	8 (22)	0.62
Fortified food consumer, No. (%)		28 (93)	33 (89)	0.87
Dietary intakes^a^	**PRI**^**c**^			
Energy, MJ/day	**6.400**	6.676 (6.118, 7.234)	6.746 (6.088, 7.404)	0.87
Dietary folate equivalents, μg/day	**270**	276 (234, 317)	265 (232, 299)	0.66
Vitamin B12, μg/day	**3.5**	4.4 (3.6, 5.2)	4.1 (3.5, 4.6)	0.55
Riboflavin, mg/day	**1.4**	1.7 (1.4, 2.0)	1.6 (1.4, 1.8)	0.49
Vitamin B6, mg/day	**1.4**	1.6 (1.5, 1.8)	1.6 (1.4, 1.8)	0.73
Biomarker status^b^	**cut-off**^**c**^			
RBC folate, nmol/L	**> 309**	595 (502, 688)	662 (513, 810)	0.45
Serum B12, pmol/L	**> 172**	511 (418, 605)	529 (460, 598)	0.75
Riboflavin, EGRac^d^	**< 1.40**	1.48 (1.38, 1.58)	1.44 (1.37, 1.52)	0.54
Plasma homocysteine, μmol/L	**< 10.7**	6.9 (6.1, 7.7)	6.9 (5.8, 8.0)	0.94

Data presented as mean (95% CI), unless otherwise indicated. Differences between groups were assessed using an independent *t* test (continuous variables) or chi-square test (categorical variables). Statistically significant difference *p* < .05

^a^Children who provided dietary intake data *n* = 48 (placebo *n* = 21; FA *n* = 27)

^b^Children who provided a blood sample *n* = 33 (placebo *n* = 16; FA *n* = 17)

^c^Population reference intakes from EFSA (2017) [24] and biomarker reference ranges from Kerr et al. (2009) [26]

^d^EGRac, erythrocyte glutathione reductase activation coefficient (biomarker of riboflavin status; a higher EGRac ratio indicates lower riboflavin status). Suboptimal riboflavin status is indicated by an EGRac value of 1.30–1.40 whereas ≥ 1.40 is considered deficient

### Effects of maternal FA intervention on child cognitive performance

**Fig. 2:**
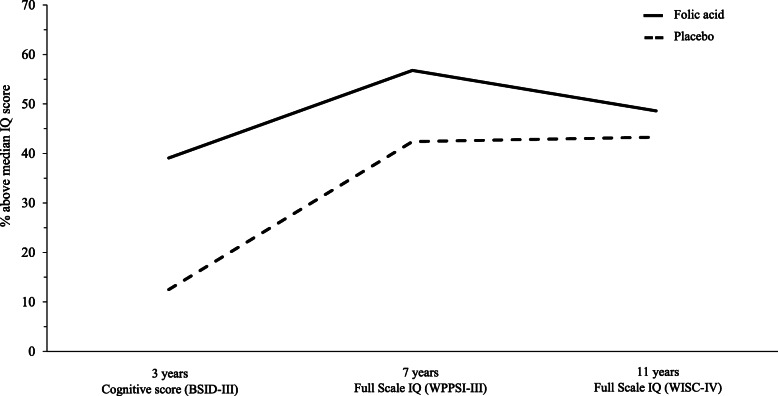
Percentage of FASSTT Offspring participants at 3, 7 and 11 years achieving above average cognitive performance by treatment allocation of the mother during pregnancy. In children at 3 years, cognitive performance was assessed by the BSITD-III test, at 7 years by the WPPSI-III test and at 11 years by the WISC-IV test. Values are percentage of children at 3, 7 or 11 years, by treatment group of the mother, who achieved above the median cognitive score for children at that age (relative to those who scored at and below the median score); median scores are 10.0 in the BSITD-III test, 23.0 in the WPPSI-III test and 102.5 in the WISC-IV test. Total participants numbers at each age are 3 years, *n* = 39; 7 years, *n* = 70; and 11 years, *n* = 68. 71.4% of the children from FA-treated mothers who had cognitive scores above the median at 11 years also scored above the median at 7 years and Full Scale IQ scores at these time points were significantly correlated (*r* = 0.463; *p* < 0.0001)

Full Scale IQ, composite domains and subtest scores from FASSTT Offspring trial participants at 11 years are presented in Table 3 and Additional File 1: Table S2. The children of mothers randomised to FA compared with placebo during pregnancy scored significantly higher in two Processing Speed tests, i.e. symbol search (+ 2.9 points) and cancellation (+ 11.3 points), whereas the positive effect of prenatal FA on Verbal Comprehension was significant in girls only (+ 6.5 points) (Table 3). At all FASSTT Offspring Trial time-points, as currently assessed at 11 years and previously at 3 and 7 years, greater proportions of children from FA-treated mothers compared with placebo had cognitive scores above the median value of 10.0 (in the BSITD-III test at 3 years), 23.0 (in the WPPSI-III test at 7 years) and 102.5 (in the WISC-IV test at 11 years), albeit by 11 years, the effect of prenatal FA appeared to have diminished somewhat (Fig. 2). Of note, 71.4% of the children from FA-treated mothers who had cognitive scores above the median at 11 years also scored above the median at 7 years and the Full Scale IQ scores at these time points were significantly correlated (*r* = 0.463; *p* < 0.0001).

**Table 3:** WISC-IV cognitive assessment and MEG brain imaging in FASSTT Offspring trial participants

	Placebo (*n* = 31)	Folic acid (*n* = 37)	Difference	*p* value^a^	*p* value^b^
**WISC-IV assessment**					
**Full Scale IQ**	101.5 (96.9, 106.0)	102.8 (99.5, 106.1)	1.3 (− 4.1, 6.7)	0.63	0.85
Boys	103.2 (95.7, 110.7)	101.3 (95.6, 107.0)	1.9 (− 10.9, 7.1)	0.66	0.61
Girls	99.9 (93.8, 106.1)	103.8 (99.5, 108.1)	3.8 (− 3.2, 10.8)	0.28	0.44
**Verbal Comprehension**	95.0 (90.9, 99.1)	97.5 (94.6, 100.3)	2.5 (− 2.4, 7.3)	0.31	0.45
Boys	97.9 (89.9, 105.9)	95.2 (90.5, 99.9)	2.7 (− 11.6, 6.1)	0.53	0.48
Girls	92.4 (88.5, 96.3)	99.0 (95.2, 102.7)	6.5 (1.2, 11.8)	0.02	0.03
**Perceptual Reasoning**	106.3 (101.4, 111.2)	104.5 (100.9, 108.2)	1.8 (− 7.6, 4.0)	0.54	0.45
Boys	107.3 (100.3, 114.3)	104.1 (97.5, 110.6)	3.2 (− 12.3, 5.9)	0.47	0.51
Girls	105.5 (98.0, 113.0)	104.9 (100.2, 109.5)	0.6 (− 8.7, 7.5)	0.87	0.85
**Working Memory**	96.9 (92.3, 101.5)	98.4 (85.5, 101.3)	1.5 (− 3.6, 6.6)	0.55	0.76
Boys	98.2 (92.1, 104.3)	98.4 (93.1, 103.7)	0.2 (− 7.5, 7.8)	0.96	0.97
Girls	95.8 (88.6, 103.1)	98.4 (94.8, 102.0)	2.6 (− 4.6, 9.8)	0.47	0.74
**Processing Speed**	104.2 (98.9, 109.6)	109.2 (105.9, 112.5)	4.9 (− 1.0, 10.9)	0.10	0.16
Boys	102.3 (94.0, 110.5)	108.4 (102.6, 114.3)	6.1 (− 3.5, 15.8)	0.20	0.21
Girls	105.9 (98.2, 113.7)	109.7 (105.4, 113.9)	3.7 (− 4.3, 11.7)	0.35	0.64
Coding subtest	47.9 (44.5, 51.3)	49.0 (46.6, 51.4)	1.1 (− 2.7, 5.0)	0.78	0.73
Symbol search subtest	24.6 (22.3, 26.9)	27.5 (26.2, 28.8)	2.9 (0.3, 5.5)	0.03	0.03
Cancellation subtest	83.7 (75.8, 91.6)	95.0 (89.6, 100.3)	11.3 (2.5, 20.1)	0.05	0.04
**MEG band power in response to the language paradigm**^**c**^					
Broad [1–48 Hz]^d^	48.9 (14.6, 83.2)	32.9 (6.7, 59.0)	16.1 (− 56.6, 24.5)	0.43	0.41
Theta [4–8 Hz]	90.8 (38.1, 143.4)	65.3 (28.7, 102.0)	25.5 (− 84.9, 34.0)	0.36	0.21
Mu [8–12 Hz]	53.9 (26.1, 81.6)	39.2 (15.1, 63.3)	14.6 (− 49.8, 20.5)	0.39	0.46
Beta [13–30 Hz]	26.4 (2.7, 50.1)	109.6 (52.4, 166.8)	83.2 (15.0, 151.4)	0.02	0.01
Low Gamma [30–48 Hz]	40.5 (− 7.6, 88.5)	45.3 (16.0, 74.5)	4.8 (− 46.5, 56.1)	0.49	0.39
High Gamma [49–70 Hz]	30.1 (− 4.4, 64.6)	100.8 (48.6, 153.0)	70.7 (6.5, 135.0)	0.04	0.04

Data presented as mean (95% CI), unless otherwise indicated. Differences between groups were analysed by ^a^independent *t* test or ^b^ANCOVA adjusting for covariates: Multiple Deprivation Measure and child’s sex. Statistically significant difference *p* < .05

^c^MEG analysis completed in a subset of children: placebo, *n* = 14; folic acid, *n* = 19. Differences between groups were analysed by ^a^independent *t* test or ^b^ANCOVA adjusting for child’s sex

^d^Broad band [1–48 Hz] is comprised of Theta, Mu, Beta and Low Gamma bands

### Effects of maternal FA intervention on child brain function response

MEG brain imaging was offered to all the participants in the trial, but owing to practical considerations (including the requirement to undertake a 2-h return journey to our NIFBM centre), not all children participated in this component. Of the 68 mother-child pairs in the current follow-up study, 33 children also completed the MEG assessment. Maternal characteristics measured during pregnancy in this sub-sample were similar to those of the total sample of FASSTT trial participants (Additional File 1: Table S3). MEG assessment of neuronal responses to a language task showed increased power at the Beta (13–30 Hz) and High Gamma (49–70 Hz) bands in children from FA-supplemented mothers compared to placebo, indicating predominant involvement of local neurons, and in turn, more efficient semantic processing of language (Table 3). Given that the MEG brain imagining was conducted only in a subset of children (i.e. 49% of child participants), the MEG results were not analysed by sex.

In addition, power at the Beta band in children was significantly correlated with maternal serum and RBC folate concentrations at the 36th GW of pregnancy (*r* = 0.439, *p* = 0.02; *n* = 33 and *r* = 0.393, *p* = 0.03; *n* = 32, respectively). Furthermore, when maternal serum folate at the 36th GW was dichotomised into lower and higher status categories based on the median value, higher maternal folate was associated with significantly higher power at the Beta (mean difference 90.7 points, 95% CI 22.5 to 158.9, *p* = 0.002), but not High Gamma (14.8, − 85.9 to 56.3, *p* = 0.99), band in the child at 11 years, after adjustment for the child’s sex and socioeconomic status. Similar correlations for cord blood folate status with MEG were found, specifically, serum folate was significantly associated with power at the Beta band (*r* = 0.439, *p* = 0.03; *n* = 26). Likewise, in children with higher cord blood folate, we found significantly higher power at the Beta band (65.0, 95% CI − 10.4 to 140.4, *p* = 0.05) at 11 years.

## Discussion

We examined the effect of FA intervention in trimesters 2 and 3 of pregnancy in women taking FA as recommended in the first trimester, and provide the first randomised trial evidence that continued FA supplementation (400 μg/day) throughout pregnancy can influence cognitive performance and brain function of the child up to 11 years of age. The results not only provide a further follow-up from the FASSTT Offspring study to reinforce our previous findings of cognitive benefits in these children at 3 and 7 years but provide the first MEG brain imaging evidence that FA supplementation in pregnancy can impact brain function in the child.

Our results, reporting the third follow-up of mother-child pairs from the FASSTT trial, show that the 11-year-old children of mothers randomised to FA compared with placebo during trimesters 2 and 3 of pregnancy scored significantly higher in specified cognitive IQ domains of the WISC-IV assessment, namely, two Processing Speed tests, i.e. symbol search (by 2.9 points) and cancellation (by 11.3 points), and Verbal Comprehension (by 6.5 points) in girls. The results are entirely consistent with our earlier findings in these children at a younger age, showing higher scores in overall cognition at 3 years and in verbal IQ at 7 years in the offspring of FA-treated mothers in pregnancy [11]. Here we show that at all time-points up to age 11 years, greater proportions of children from FA-treated mothers, compared with placebo, had cognitive scores above the median values of 10.0 in the BSITD-III test (at 3 years), 23.0 in the WPPSI-III test (at 7 years) and 102.5 in the WISC-IV test (at 11 years). The FASSTT Offspring trial thus provides more robust evidence of the role of maternal folate status in offspring cognitive function compared with previous observational studies that have reported positive associations between FA use in early pregnancy (as retrospectively reported by mothers) or maternal folate concentrations, and cognitive performance in the child [6, 28, 29]. The role of maternal folate beyond the first trimester of pregnancy has been far less frequently investigated in previous studies, but one notable study found that higher plasma folate at the 30th gestational week was associated with better cognitive performance in over 500 children aged 9–10 years in South India [8]. Our results are thus consistent with previous observations, but add considerably to the evidence, and indicate that FA intervention throughout pregnancy will have beneficial effects on cognitive performance in the child up to 11 years. Of note, folate intake and status of the children were considered in this study and found to be similar in both study groups, with dietary folate comparing favourably with reference values for this age group [24] and biomarker concentrations in good agreement with those reported in children from Norway [25], UK [26] and USA [27].

The current sex-specific findings, showing a positive effect of prenatal FA on verbal IQ in 11-year-old girls (by 6.5 points), but not in boys, are not entirely unexpected given existing evidence of sex differences in brain activity patterns associated with cognition and behaviour and in functional brain development in infancy and early childhood [4]. Also, between 3 and 60 months, females are reported to exhibit a higher rate of myelination than do males in the genu of the corpus callosum, in left frontal and left temporal white matter and in the right optic radiation [30]. Furthermore, in previous epigenetic analysis of cord samples from these children at birth, we investigated candidate genes related to brain development or function and reported significant DNA methylation effects in *IGF2* in girls, but not in boys, arising from maternal FA intervention [31], perhaps offering a biological basis for the sex-differences found here in the relationship of prenatal FA with cognitive outcomes in childhood. Sex differences in brain development, however, likely reflect a dynamic interplay of many biological (e.g. prenatal and neonatal hormone production and direct sex chromosome effects) and other mechanisms [4]. In any case, this aspect requires further investigation.

In addition to using standardised IQ tests to measure child cognition, we applied magnetoencephalographic brain imaging for the first time in a study of this kind. In broad support of the cognitive outcomes, the neuronal activity assessments using MEG indicated more efficient semantic processing of language in the 11-year-old child as a result of prenatal intervention with FA. Specifically, the increased power at Beta and High Gamma bands in response to the language task in children from the FA group suggest more active engagement of local neuronal networks. Previously, increased power at the High Gamma band using MEG was reported as a result of intensive computer-based training [32]. The fact that we observed similar band power patterns in response to the language task in children of FA-supplemented mothers to those reported in children with enhanced cognition as a consequence of intensive training suggests that the current MEG findings are clinically relevant.

The finding of FA-related effects in language processing as assessed by MEG is broadly in line with the higher verbal comprehension scores in the current WISC-IV assessment at 11 years and our earlier findings of higher verbal IQ in these children at 7 years in response to prenatal FA. Likewise, a recent study reported greater cerebral cortical thickness in children aged 8–18 years who were exposed prenatally to population-wide FA fortification compared to those born before the introduction of this mandatory policy in the USA [33], whilst in the Generation R Study, maternal folate deficiency diagnosed after the first trimester was associated with reduced brain volume (measured using magnetic resonance imaging) in 6–8 year old Dutch children [9]. Thus, our randomised trial results, together with the available observational evidence, are consistent in indicating that prenatal maternal FA supplementation affects neurocognitive development and may have a specific role in semantic processing of language up to 11 years. In addition, there may be clinical implications of our findings for later life in that the achievement of full cognitive potential of the child is considered paramount for future academic attainment, and higher intelligence in childhood is associated with better cognitive reserves in adulthood that could potentially delay cognitive decline in later life [34, 35]. Future MEG studies might investigate the functional neural connectivity patterns within emerging neural networks, both in resting-state and under task conditions, that support developing language and cognition.

Although the precise mechanism explaining the effect of FA during pregnancy on neurodevelopment of the child is unknown, it must in some way involve the essential role of folate in one-carbon metabolism, encompassing a complex network of interdependent pathways required for a number of biological processes that could impact neurodevelopment, including myelination, neurotransmitter synthesis and methylation [36]. It is also possible that folate-related epigenetic changes via DNA methylation are involved [3]. In previous epigenetic analysis of cord blood samples in these children as newborns, using a candidate gene approach we showed that prenatal FA throughout pregnancy resulted in significant effects in DNA methylation in specific genes linked with brain development or function (*IGF2*, *BDNF* and the widely dispersed retrotransposon, *LINE-1*) [31], and in epigenome-wide screening of the same samples, we identified a novel mechanism for folate-dependent regulation of the *ZFP57* gene [37].

The main strength of this study is the research design, involving a follow-up study in children of mothers who had participated in a randomised trial in pregnancy, which enabled the demonstration of a causative link between prenatal FA and subsequent neurocognitive development in the child. The use in a sub-set of children of the neuroimaging technique, MEG, objectively investigated the effects of FA intervention in pregnancy on brain function in childhood. Furthermore, this is the third follow-up of the FASSTT Offspring cohort (with previous investigations at age 3 and 7 years), providing the opportunity to track cognitive development in childhood, and the broad agreement in results at all time-points provides some degree of internal validation to the findings. In addition, a range of factors previously reported to be associated with cognitive performance, and thus potential confounders in the relationship between prenatal FA and offspring intelligence, were considered [23, 38]. However, this study also has limitations, the most notable of which is the relatively small sample size. Although every effort was made to maximise the participation rate from the original FASSTT trial (*n* = 119), the final sample of 68 mother-child pairs may have lacked sufficient statistical power to detect small differences in some components of the WISC-IV cognitive test. Also paternal factors, recently emerging as potentially important for child neurocognitive development, were not considered.

## Conclusions

In summary, our findings in the children of participants in a randomised trial in pregnancy add considerably to the existing evidence from observational studies that have linked maternal folate status in pregnancy with neurocognitive outcomes in the child. We examined the effect of FA intervention in trimesters 2 and 3 of pregnancy (in women taking FA as recommended in the first trimester), and provide the first randomised trial evidence that continued FA supplementation (400 μg/day) throughout pregnancy can influence cognitive performance and brain function of the child up to 11 years of age. In addition to using standardised IQ tests to measure child cognition, we applied magnetoencephalographic brain imaging for the first time in a study of this kind, and the findings not only reinforce our previous findings of cognitive benefits in these children at 3 and 7 years but provide new evidence of a specific role of prenatal FA in semantic processing of language. The totality of evidence has important impacts in informing new policy and clinical practice in relation to FA use in pregnancy. Whereas current recommendations for NTD prevention in the UK and most countries worldwide advise mothers to take FA supplements from before conceiving until the end of the 12th gestational week only, our findings suggest that continued FA intervention in pregnancy beyond the early period required to prevent NTD is beneficial to neurocognitive development in the child. In a wider context, we demonstrate the application of MEG as a non-invasive tool in paediatric research to objectively assess functional brain activity in response to nutrition and other interventions.

## Supplementary Information

**Additional file 1: Methods.** Supplementary details in relation to magnetoencephalographic brain imaging. **Table S1**. Maternal characteristics during pregnancy in all FASSTT trial participants and in the sample whose children completed the FASSTT Offspring trial at 11 years. **Table S2**. Full Scale IQ, composite and subtest scores for the WISC-IV cognitive assessment in FASSTT Offspring trial participants. **Table S3.** Maternal characteristics during pregnancy in all FASSTT trial participants and in the sub-sample whose children completed the MEG assessment in the FASSTT Offspring trial.

## Data Availability

Data from this study are held in full compliance with Ulster University’s Research Governance and Ethics Policy for Human Research (2018) https://internal.ulster.ac.uk/research/office/rofficeeg.php, which in turn is fully aligned with the UK’s Data Protection Act 2018. The participants of FASSTT and FASSTT Offspring Trials did not provide consent for sharing their data publicly. Data are available from the Research Governance of Ulster University (UK) for researchers who meet the criteria for access to confidential data. Please address requests to Mr. Nick Curry, Head of Research Governance at Ulster University at n.curry@ulster.ac.uk.

## Abbreviations

ANCOVA: Analysis of covariance
BMI: Body mass index
BSITD-III: Bayley’s Scales of Infant and Toddler Development 3rd UK Edition
FA: Folic acid
FASSTT: Folic Acid Supplementation during the Second and Third Trimesters
GW: Gestational weeks
IQ: Intelligence quotient
MTHFR: Methylenetetrahydrofolate reductase
NTDs: Neural tube defects
RBC: Red blood cell
RCT: Randomised controlled trial
SPSS: Statistical Package for the Social Sciences software
WISC-IV: Wechsler Intelligence Scale for Children 4th UK edition
WPPSI-III: Wechsler Preschool and Primary Scale of Intelligence 3rd UK edition
