# Supplementary material for: Effects of maternal folic acid supplementation during the second and third trimesters of pregnancy on neurocognitive development in the child: an 11-year follow-up from a randomised controlled trial
Source: BMC Med. 2021 Mar 10;19:73. doi: 10.1186/s12916-021-01914-9 (PMC7945668; doi:10.1186/s12916-021-01914-9)
Supplement: Supplementary file 1 — Additional file 1: Methods. Supplementary details in relation to magnetoencephalographic brain imaging. Table S1. Maternal characteristics during pregnancy in all FASSTT trial participants and in the sample whose children completed the FASSTT Offspring trial at 11 years. Table S2. Full Scale IQ, composite and subtest scores for the WISC-IV cognitive assessment in FASSTT Offspring trial participants. Table S3. Maternal characteristics during pregnancy in all FASSTT trial participants and in the sub-sample whose children completed the MEG assessment in the FASSTT Offspring trial. [file 12916_2021_1914_MOESM1_ESM.docx]

**ADDITIONAL FILE 1**

**Methods.** Supplementary details in relation to magnetoencephalographic brain imaging

**Table S1.** Maternal characteristics during pregnancy in all FASSTT trial participants and in the sample whose children completed the FASSTT Offspring trial at 11 years

**Table S2.** Full Scale IQ, composite and subtest scores for the WISC-IV cognitive assessment in FASSTT Offspring trial participants

**Table S3.** Maternal characteristics during pregnancy in all FASSTT trial participants and in the sub-sample whose children completed the MEG assessment in the FASSTT Offspring trial

**METHODS.** Supplementary details in relation to magnetoencephalographic brain imaging

**Semantic Language Paradigm [comprising auditorily presented congruent and incongruent sentences]**

In the congruent condition, the sentences ended with a word that completed the sentence in a semantically valid way i.e. ‘the climbers reached the top of the mountain’. For the incongruent condition, sentences ended with a word which was unexpected in the sentential context, resulting in a semantic violation word, i.e. ‘the climbers reached the top of the tulip’. A matched set of 60 correct and 60 incorrect sentences were constructed for the experimental condition. No specific voice changes marked the final word for either correct or incorrect sentences. The order of the sentences was randomised for each participant so that no subject heard the same sentence more than once. The language task was presented to each participant in a block, which consisted of 4 sets of 30 sentences, resulting in a total of 120 sentences.

**Elekta Neuromag 306-channel MEG system: Operating conditions**

Children’s averaged neural responses across the stimulus types, provide a neurophysiological proxy for semantic language ability, as indexed by the power of the MEG signal in different frequency bands, and where higher language ability is linked to greater differences in neural activity between the congruent and incongruent trials. Changes in power within the beta-frequency band, in particular, have been linked to the magnitude of the semantic congruency effect [Wang et al., 2012; Ghosh Hajra et al., 2018]. The band power in response to the language paradigm was obtained from 116 brain regions and averaged within six standard spectral bands: Big band [1 –48Hz], Theta [4–8Hz], Mu [8–12Hz], Beta [13–30Hz], Low Gamma [30–48Hz] and High Gamma [49–70Hz].

The signal was recorded with a sampling rate of 1 kHz using 204-planar gradiometers and 102-magnetometers. The Neuromag software MaxFilter 2.2 (Elekta Neuromag, Stockholm, Sweden) that implements spatiotemporal signal space separation (tSSS) and movement correction was used to pre-process the signal and to compensate for any head movement. This method reduces the effect of interference and disturances to a larger extent, and separates magnetic signals within the brain from those coming from outside the brain. Finally, the signal was low pass-filtered at 100 Hz and stored for analysis. The analysis of the signals and the time-window of interest was 300-500 ms to capture N400 component which has previously been linked to language processing capabilities. The experiment was computerised through MATLAB (Mathworks, Natick, USA).

**References**

Wang L, Jensen O, van den Brink D, Weder N, Schoffelen J‐M, Magyari L, Hagoort P, Bastiaansen, M. Beta oscillations relate to the N400m during language comprehension. Hum Brain Mapp. 2012;33:2898-2912.

Ghosh Hajra S, Liu CC, Song X, Fickling SD, Cheung T, D'Arcy R. Multimodal characterization of the semantic N400 response within a rapid evaluation brain vital sign framework. J Transl Med. 2018;16:151.

**Table S1.** Maternal characteristics during pregnancy in all FASSTT trial participants and in the sample whose children completed the FASSTT Offspring trial at 11 years

|  | FASSTT trial (*n* = 119) | |  | | FASSTT Offspring trial (*n* = 68) | |  |
| --- | --- | --- | --- | --- | --- | --- | --- |
|  | Placebo (*n* = 60) | Folic acid (*n* = 59) | *p* value^a^ |  | Placebo (*n* = 31) | Folic acid (*n* = 37) | p value^a^ |
| **Maternal characteristics** |  |  |  |  |  |  |  |
| Maternal age, y | 28.0 (26.9, 29.2) | 29.2 (28.2, 30.3) | 0.12 |  | 28.1 (26.6, 29.6) | 29.7 (28.5, 30.8) | 0.09 |
| BMI, kg/m^2^ | 24.3 (23.4, 25.3) | 25.0 (23.7, 26.3) | 0.39 |  | 25.2 (23.7, 26.7) | 25.5 (23.6, 27.3) | 0.83 |
| Gestation at labour, wk | 40.1 (39.8, 40.5) | 39.8 (39.4, 40.1) | 0.12 |  | 40.2 (39.6, 40.7) | 39.8 (39.4, 40.2) | 0.27 |
| Socioeconomic status (MDM)^b^ | 20.4 (17.2, 23.5) | 18.4 (15.8, 21.0) | 0.33 |  | 21.9 (17.0, 26.8) | 18.7 (15.3, 22.0) | 0.26 |
| Serum folate, nmol/L |  |  |  |  |  |  |  |
| Preintervention (14^th^ GW) | 45.7 (40.2, 41.2) | 47.0 (41.5, 52.4) | - |  | 50.5 (43.1, 57.9) | 49.9 (42.8, 57.1) | - |
| Postintervention (36^th^ GW) | 19.5 (15.0, 23.9) | 48.2 (42.1, 54.4) | - |  | 22.4 (15.9, 28.8) | 52.9 (45.8, 60.0) | - |
| Response | -26.1 (-31.2, -21.0) | 0.9 (-5.7, 7.5) | <0.001 |  | -28.2 (-34.4, -20.5) | 2.9 (-5.9, 11.2) | <0.001 |
| Plasma homocysteine, µmol/L |  |  |  |  |  |  |  |
| Preintervention | 6.7 (6.0, 7.3) | 6.3 (5.9, 6.6) | - |  | 6.1 (5.6, 6.6) | 6.1 (5.6, 6.6) | - |
| Postintervention | 7.6 (7.0, 8.2) | 6.4 (6.0, 6.8) | - |  | 7.2 (6.5, 7.9) | 6.3 (5.8, 6.8) | - |
| Response | 1.0 (0.4, 1.5) | 0.1 (-0.2, 0.5) | <0.01 |  | 1.1 (0.4, 1.7) | 0.2 (-0.1, 0.5) | 0.01 |
| **Neonatal characteristics** |  |  |  |  |  |  |  |
| Gestational age, wk | 40.2 (39.8, 40.5) | 39.8 (39.4, 40.1) | 0.10 |  | 40.2 (39.6, 40.7) | 39.8 (39.4, 40.2) | 0.27 |
| Birth weight, g | 3476 (3360, 3593) | 3443 (3306, 3581) | 0.71 |  | 3503 (3329, 3677) | 3461 (3290, 3633) | 0.73 |
| Birth length, cm | 50.9 (50.2, 51.6) | 50.7 (50.1, 51.3) | 0.66 |  | 51.1 (50.2, 52.0) | 50.9 (50.1, 51.7) | 0.76 |
| Head circumference, cm | 34.7 (34.3, 35.0) | 34.7 (34.3, 35.0) | 0.99 |  | 34.5 (34.0, 35.0) | 34.6 (34.1, 35.1) | 0.82 |

Data presented as mean (95% CI).

^a^Time x treatment interaction (within-between repeated measures ANOVA) comparing the effect of treatment with placebo over time. Statistically significant difference *p* < 0.05.

^b^Northern Ireland Multiple Deprivation Measure (MDM) 2010. This is a measure of socioeconomic area-based deprivation and comprises 7 domains to each developed to measure a distinct form or type of deprivation: income to employment to health to education to proximity to services to living environment to and crime.

**Table S2.** Full Scale IQ, composite and subtest scores for the WISC-IV cognitive assessment in FASSTT Offspring trial participants at 11 years

|  | Placebo (*n* = 31) | Folic Acid (*n* = 37) | Difference | *p* value^a^ | *p* value^b^ |
| --- | --- | --- | --- | --- | --- |
| **Full Scale IQ** | 101.5 (96.9, 106.0) | 102.8 (99.5, 106.1) | 1.3 (-4.1, 6.7) | 0.63 | 0.85 |
| **Verbal Comprehension** | 95.0 (90.9, 99.1) | 97.5 (94.6, 100.3) | 2.5 (-2.4, 7.3) | 0.31 | 0.45 |
| Similarities | 21.3 (19.4, 23.3) | 22.8 (21.2, 24.3) | 1.4 (-2.1, 5.0) | 0.42 | 0.48 |
| Vocabulary | 35.0 (32.3, 37.6) | 33.3 (31.2, 35.3) | 1.7 (-6.5, 3.1) | 0.33 | 0.23 |
| Comprehension | 22.3 (20.8, 23.8) | 22.4 (21.3, 23.4) | 0.1 (-2.5, 2.7) | 0.78 | 0.71 |
| Information | 17.0 (16.0, 18.1) | 17.9 (17.1, 18.7) | 0.9 (-0.3, 2.1) | 0.23 | 0.27 |
| Word Reasoning | 16.5 (15.7, 17.4) | 16.2 (15.4, 16.9) | 0.3 (-1.4, 0.7) | 0.53 | 0.40 |
| **Perceptual Reasoning** | 106.3 (101.4, 111.2) | 104.5 (100.9, 108.2) | 1.8 (-7.6, 4.0) | 0.54 | 0.45 |
| Block design | 38.9 (34.5, 43.3) | 36.0 (33.0, 39.0) | 2.9 (-7.8, 1.9) | 0.18 | 0.14 |
| Picture concepts | 18.6 (17.6, 19.7) | 19.2 (18.3, 20.0) | 0.6 (-0.7, 1.8) | 0.80 | 0.77 |
| Matrix reasoning | 23.1 (21.5, 24.7) | 23.1 (21.8, 24.5) | 0.0 (-1.9, 2.0) | 0.47 | 0.36 |
| Picture completion | 23.0 (21.3, 24.7) | 24.4 (23.0, 25.7) | 1.4 (-0.7, 3.5) | 0.27 | 0.26 |
| **Working Memory** | 96.9 (92.3, 101.5) | 98.4 (85.5, 101.3) | 1.5 (-3.6, 6.6) | 0.55 | 0.76 |
| Digit span | 16.6 (15.3, 17.9) | 15.9 (15.0, 16.9) | 0.7 (-2.2, 0.9) | 0.23 | 0.20 |
| Letter-number sequencing | 16.9 (15.9, 18.0) | 17.6 (16.8, 18.3) | 0.6 (-0.6, 1.8) | 0.60 | 0.77 |
| Arithmetic | 24.7 (23.4, 26.0) | 24.5 (23.5, 25.5) | 0.2 (-1.8, 1.4) | 0.59 | 0.45 |
| **Processing Speed** | 104.2 (98.9, 109.6) | 109.2 (105.9, 112.5) | 4.9 (-1.0, 10.9) | 0.10 | 0.16 |
| Coding | 47.9 (44.5, 51.3) | 49.0 (46.6, 51.4) | 1.1 (-2.7, 5.0) | 0.78 | 0.73 |
| Symbol search | 24.6 (22.3, 26.9) | 27.5 (26.2, 28.8) | 2.9 (0.3, 5.5) | 0.03 | 0.03 |
| Cancellation | 83.7 (75.8, 91.6) | 95.0 (89.6, 100.3) | 11.3 (2.5, 20.1) | 0.05 | 0.04 |

Data presented as mean (95% CI). Differences between groups were analysed by ^a^independent t-test or ^b^ANCOVA adjusting for covariates: Socioeconomic status (MDM) and child’s sex. Statistically significant difference p<0.05.

**Table** **S3****.** Maternal characteristics during pregnancy in all FASSTT trial participants and in the sub-sample whose children completed the MEG assessment in the FASSTT Offspring trial

|  | FASSTT trial (total sample; *n*=119) | | |  | FASSTT Offspring trial (sub-sample; *n*=33) | | |
| --- | --- | --- | --- | --- | --- | --- | --- |
|  | Placebo (*n* = 60) | Folic acid (*n* = 59) | *p* value^1^ |  | Placebo (*n* = 14) | Folic acid (*n* = 19) | *p* value^1^ |
| Maternal age, y | 28.0 (26.9 to 29.2) | 29.2 (28.2 to 30.3) | 0.12 |  | 28.9 (26.0 to 31.9) | 30.2 (28.7 to 31.6) | 0.39 |
| BMI, kg/m^2^ | 24.3 (23.3 to 25.3) | 25.0 (23.7 to 26.3) | 0.39 |  | 23.7 (21.8 to 25.7) | 25.2 (22.8 to 27.7) | 0.35 |
| Gestation at labor, wk | 40.1 (39.8 to 40.5) | 39.8 (39.4 to 40.1) | 0.12 |  | 40.1 (39.3 to 41.0) | 39.5 (38.9 to 40.0) | 0.67 |
| Socioeconomic status (MDM)^b^ | 20.4 (17.2 to 23.5) | 18.4 (15.8 to 21.0) | 0.33 |  | 20.7 (12.4 to 29.0) | 17.2 (13.7 to 20.6) | 0.35 |
| Serum folate, nmol/L |  |  |  |  |  |  |  |
| Preintervention | 45.7 (40.2 to 41.2) | 47.0 (41.5 to 52.4) | **-** |  | 49.1 (37.6 to 60.0) | 56.2 (46.3 to 66.1) | **-** |
| Postintervention | 19.5 (15.0 to 23.9) | 48.2 (42.1 to 54.4) | **-** |  | 25.7 (12.6 to 38.8) | 56.9 (45.2 to 68.7) | **-** |
| Response | -26.1 (-31.2 to -21.0) | 0.9 (-5.7 to 7.5) | <0.001 |  | -23.4 (-33.3 to -13.4) | 0.7 (-9.2 to 10.7) | <0.01 |
| Plasma homocysteine, µmol/L |  |  |  |  |  |  |  |
| Preintervention | 6.7 (6.0 to 7.3) | 6.3 (5.9 to 6.6) | - |  | 5.6 (5.1 to 6.2) | 5.9 (5.4 to 6.4) | - |
| Postintervention | 7.6 (7.0 to 8.2) | 6.4 (6.0 to 6.8) | - |  | 7.0 (6.0 to 8.0) | 5.9 (5.4 to 6.5) | - |
| Response | 1.0 (0.4 to 1.5) | 0.1 (-0.2 to 0.5) | <0.01 |  | 1.4 (0.6 to 2.2) | 0.0 (-0.4 to 0.4) | <0.01 |

Data presented as mean (95% CI).

^a^Time x treatment interaction (within-between repeated measures ANOVA) comparing the effect of treatment with placebo over time. Statistically significant difference p<0.05.

^b^Northern Ireland Multiple Deprivation Measure (MDM) 2010. This is a measure of socioeconomic area-based deprivation and comprises 7 domains to each developed to measure a distinct form or type of deprivation: income to employment to health to education to proximity to services to living environment to and crime.
